# Expression Levels and Localizations of DVL3 and sFRP3 in Glioblastoma

**DOI:** 10.1155/2017/9253495

**Published:** 2017-10-19

**Authors:** Anja Kafka, Davor Tomas, Mirna Lechpammer, Tea Gabud, Leo Pažanin, Nives Pećina-Šlaus

**Affiliations:** ^1^Laboratory of Neuro-oncology, Croatian Institute for Brain Research, School of Medicine, University of Zagreb, Salata 12, Zagreb, Croatia; ^2^Department of Biology, School of Medicine, University of Zagreb, Salata 3, Zagreb, Croatia; ^3^Department of Pathology, School of Medicine, University of Zagreb, Salata 10, 10000 Zagreb, Croatia; ^4^University Hospital “Sisters of Charity”, Vinogradska 29, 10000 Zagreb, Croatia; ^5^Department of Pathology & Laboratory Medicine, University of California, Davis Medical Center, 4400 V Street, Sacramento, CA 95817, USA

## Abstract

The expression patterns of critical molecular components of Wnt signaling, sFRP3 and DVL3, were investigated in glioblastoma, the most aggressive form of primary brain tumors, with the aim to offer potential biomarkers. The protein expression levels and localizations in tumor tissue were revealed by immunohistochemistry and evaluated by the semiquantitative method and immunoreactivity score. Majority of glioblastomas had moderate expression levels for both DVL3 (52.4%) and sFRP3 (52.3%). Strong expression levels were observed in 23.1% and 36.0% of samples, respectively. DVL3 was localized in cytoplasm in 97% of glioblastomas, of which 44% coexpressed the protein in the nucleus. sFRP3 subcellular distribution showed that it was localized in the cytoplasm in 94% of cases. Colocalization in the cytoplasm and nucleus was observed in 50% of samples. Wilcox test indicated that the domination of the strong signal is in connection with simultaneous localization of DVL3 protein in the cytoplasm and the nucleus. Patients with strong expression of DVL3 will significantly more often have the protein in the nucleus (*P* = 6.33 × 10^−5^). No significant correlation between the two proteins was established, nor were their signal strengths correlated with epidemiological parameters. Our study contributes to better understanding of glioblastoma molecular profile.

## 1. Introduction

Glioblastoma is the most frequent and deadliest malignant brain tumor, classified as grade IV by the World Health Organization (WHO) [1, 2]. Despite recent advances in diagnosis and treatment, the prognosis and survival remain poor [3, 4] since the tumor is resistant to available therapies. Glioblastoma is the most aggressive and invasive astrocytic tumor [1, 3] that also shows great heterogeneity both genetically and morphologically. Over the past 20 years, cytogenetic and molecular genetic changes associated with the formation and progression of astrocytomas were intensively studied. The complex mechanism of gliomagenesis results from interconnection and overlapping of altered signaling pathways. At the histological level, it is difficult to distinguish between primary and secondary types of glioblastoma. However, at the molecular level, numerous differences in the frequency of mutations of particular genes have been established [3]. It is now clear that one of the most common mutations in gliomagenesis is the mutation R132H of the isocitrate dehydrogenase 1 (IDH1) gene, and it can be detected at a very early stage in diffuse astrocytoma. IDH wild-type and IDH mutant glioblastoma display different characteristics [2, 5, 6], and IDH mutant glioblastoma will also exhibit 71% of ATRX gene mutations [3].

Wnt signaling plays central roles in both the development and cancer. It regulates critical processes of normal CNS development [7–10] and is also one of the key oncogenic pathways in great number of human malignancies. In recent years, there has been increasing evidence including our own investigations that the formation of glioblastomas is, alongside other signaling pathways, also driven by Wnt signaling [11–21].

We were interested in investigating two important Wnt signaling molecules, Dishevelled 3 (DVL3) and Secreted Frizzled-related protein 3 (sFRP3), and testing the hypothesis that their expression levels were correlated with clinicopathological features and glioblastoma phenotype in order to offer potential diagnostic and prognostic biomarkers. Suppression of the Wnt signaling is necessary for the normal development of astrocytes and is mediated by the secreted Frizzled-related protein (sFRP) family [22]. sFRP family members code for proteins that can limit Wnt signaling activity. At the plasma membrane, these soluble proteins directly bind to Wnt ligands or to Frizzleds (Fz), the serpentine receptors of the pathway. Frizzleds are responsible for binding of Wnt ligands in the first place, but sFRPs can separate the ligands from the receptors and thus antagonize signaling [23–25]. In the presence of Wnt ligands, phosphorylated DVL is recruited to the plasma membrane, where it interacts with Frizzled receptors and polymerizes with other DVL molecules. Polymerization leads to GSK-3beta inactivation, resulting in dephosphorylation of many proteins, including beta-catenin [26]. Lack of Wnt signaling suppression causes stabilization of beta-catenin in cytoplasm, its transfer to the nucleus, and activation of a variety of target genes involved in cell cycle progression, which consequently leads to tumorigenesis.

Dishevelled 3 (DVL3) is located at 3q27, consists of 716 amino acids, and is also a member of a multigene family [27, 28]. According to recent studies, Dishevelleds are multifunctional phosphoproteins and key regulators that rescue cytoplasmic *β*-catenin from degradation. DVL3 can shuttle between cytoplasm and nucleus which can challenge the conventional thinking about its function and suggest that DVL3 might act differently depending on its location in the cell [29–31]. Since Dvl proteins have been attributed a central position in the Wnt signaling, their inclusion and roles in tumor formation have been under intensive investigation. However, the roles of individual Dvls and whether DVL3 overexpression is related to tumor prognosis are still poorly defined.

sFRPs are the largest family of soluble proteins known for their ability to modulate the Wnt signaling [32–34]. sFRP3, originally named FrzB (or FrzB1), is a founding member of the family, located at 2q32.1 and codes for the protein of 325 amino acids. sFRP3 is widely expressed in adult mammalian tissues [35] and generally has been attributed with an antagonistic role [36] in Wnt signaling. Contrary, when regulating cell growth and differentiation, the action of sFRP3 need not always be inhibitory [34].

Our study aims to identify the status of Wnt signaling key proteins, DVL3 and sFRP3, in human glioblastoma, and search for their connection with clinicopathological data in order to offer potential diagnostic and prognostic biomarkers.

## 2. Materials and Methods

### 2.1. Tumor Specimens

Thirty-four glioblastoma samples were collected from the University Hospital Center “Sisters of Charity” Zagreb, Croatia. The tumors were identified by magnetic resonance imaging in different cerebral regions. Selected patients had no family history of brain tumors or familial tumor syndromes, and diagnosis was made by a board-certified neuropathologist and classified according to WHO guidelines [1–3]. Recurrent tumors were found in 26.5% of patients. Available patients were also tested for the presence of IDH1 R132H and ATRX mutations. Eighteen patients were male, and 16 were female. Patient age ranged from 20 to 77 years (mean: 56.4 years; median: 59.5 years). Ethical approval was received from the Ethical Committees of Medical School University of Zagreb, University Hospital Center, Sisters of Charity (Zagreb, Croatia), and the patients gave their informed consent.

### 2.2. Immunohistochemistry

The samples were fixed in formalin, embedded in paraffin, sliced into 4 *μ*m thick sections, and mounted into capillary gap microscope slides (DakoCytomation, Denmark). Sections were immunostained using streptavidin horseradish peroxidase/DAB (Dako REAL EnVision Detection System Peroxidase/DAB+, Rabbit/Mouse, Dako, Glostrup, Denmark). Briefly, sections were deparaffinized in xylene (Kemika, Zagreb, Croatia), rehydrated in a descending ethanol dilution series (Kemika, Zagreb, Croatia), rinsed in dH_2_O for 5 min and then microwaved twice for 10 min at 700 W in retrieval solution (Dako, Glostrup, Denmark), cooled at room temperature for 15 min, and microwaved once for 5 min at 350 W to unmask the epitopes. To block endogenous peroxidase activity, cells were fixed in methanol with 3% H_2_O_2_. Nonspecific binding was blocked by incubating samples with protein block serum-free ready-to-use (Dako, Carpinteria, CA, USA) for 30 min at 4°C. Next, the primary antibodies, mouse monoclonal anti-human DVL3 (1 : 50; G-7, Santa Cruz Biotechnology Inc., Dallas, TX, USA), and rabbit polyclonal anti-human FRP3 (1 : 50; H-170, Santa Cruz Biotechnology Inc., Dallas, TX, USA) were applied for 30 min at room temperature. Slides were then washed three times in phosphate-buffered saline (PBS)/goat serum, and secondary LINK antibody was applied for 20 min at room temperature. Slides were again washed three times in PBS/goat serum and were incubated with substrate chromogen solution (Dako REAL EnVision Detection System Glostrup, Denmark) for 30 seconds. The sections were counterstained with Harris' hematoxylin. Negative controls underwent the same staining procedure but without incubating samples with the primary antibodies. The frontal cortex of a normal adult brain, human placenta, and malignant melanoma tissues were used as positive controls. Immunohistochemical staining was evaluated by assessing staining intensity by three independent and blinded observers. No expression or very weak expression was labeled as 0/+, moderate expression as ++, and strong expression as +++. Two hundred cells in a hot spot of each sample were analyzed. The slides were scanned on digital scanner (NanoZoomer 2.0RS, Hammamatsu), and for each sample, staining intensity in a well-defined area was evaluated using ImageJ (NIH, USA) program.

### 2.3. Statistical Analysis

The correlations of protein expressions and localizations between DVL3 and sFRP3 as well as their correlation to pathological and demographic features were analyzed. All individuals were analyzed for the following features: sex, age, DVL3 and sFRP3 protein expression intensities and localizations, and recurrence. Available samples were tested for IDH1 and ATRX mutations. Statistical relevance was tested with Pearson's correlations and Student's *t*-test depending of the number of cases. The distribution of protein signals on subcellular localization was tested with Mann–Whitney *U* test (also known as Wilcox rank-sum test (WRS)). This nonparametric test has null hypothesis that two groups of samples originate from the same population, contrary to the alternative hypothesis. In this case, specific population takes on higher values from the other. It can be applied on unknown distributions. Essentially, this test proves that the distribution is opposed to normal distribution. The sum of Wilcox rank was tested with the correction for continuity.

Statistical significance was set at *P* < 0.05. All statistical evaluations were performed with the SPSS statistical package 14.0 (SPSS Inc., Chicago, IL, USA).

## 3. Results

We analyzed expression levels of DVL3 and sFRP3 proteins in 34 glioblastomas with an emphasis on subcellular localizations. The tissue expression levels were determined by the semiquantitative method in the 3-stage signal strength. In order to assess signal expression levels of DVL3 and sFRP3, the immunostains were compared to expression levels of normal frontal cortex and white matter, placenta, and human malignant melanoma tissue. The levels of DVL3 expression in normal brain was evaluated as weak and localized only in the cytoplasm, and the staining of sFRP3 in normal brain showed membranous staining pattern without diffuse cytoplasmic or nuclear expression. The other positive controls showed weak or moderate expression levels of both proteins. In placenta, sFRP3 expression was confined to blood vessel endothelial cells, while decidual cells showed only occasional weak cytoplasmic staining. Perimetrium and myometrium were also positive for sFRP3 expression [37]. We demonstrated that DVL3 and sFRP3 proteins were present in glioblastoma tissue samples. The majority of tumors showed moderate levels of expression for both DVL3 (52.4%) and sFRP3 (52.3%). Furthermore, we observed that sFRP3 (11.7%) showed lower number of counted cells than DVL3 (24.5%) in the category of the weak signal, while in the category of the strong signal, sFRP3 was more frequent (36%) than DVL3 (23.1%) (Table 1).

Another parameter that was investigated was the subcellular localization of the proteins. DVL3 was localized exclusively in the cytoplasm in 53% of glioblastomas, while 44% had the protein coexpressed in the cytoplasm and nucleus. Not a single tumor showed only nuclear localization of the DVL3 protein. In 56% of cases with cytoplasmic expression, the protein was located in close proximity to the cell membrane (Table 2).

**Figure 1:**
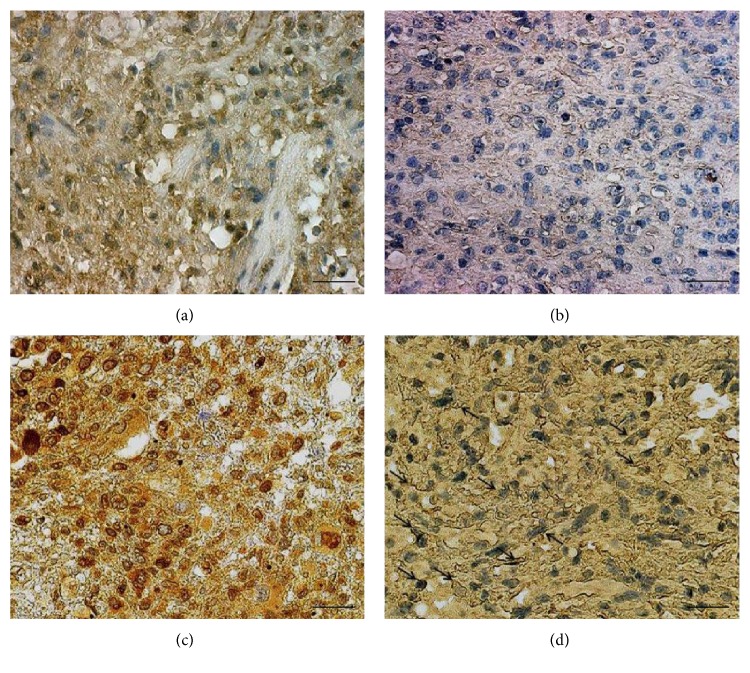
Characteristic immunohistochemical staining of DVL3 and sFRP3 expression levels. (a) Glioblastoma showing strong cytoplasmic and nuclear staining of DVL3, (b) glioblastoma showing weak cytoplasmic staining of DVL3, (c) glioblastoma showing strong cytoplasmic and nuclear staining of sFRP3, and (d) glioblastoma showing moderate cytoplasmic and membranous staining of sFRP3 (arrow). Scale bars 50 *μ*m.

The results of sFRP3 protein's subcellular distribution showed that it was localized exclusively in the cytoplasm in 44% of analysed glioblastoma samples while 50% showed simultaneous cytoplasmic and nuclear localization. Nevertheless, the signal was also detected along the cell membranes of each sample. The membranous signal was distributed in a thin line that framed the cell (Figure 1).

We also wanted to see whether there is a difference between the intensity of the signal and localization. Therefore, we divide our sample into two groups, one with weak expression and the other group with moderate and strong expression. Here, we demonstrated strong statistical differences for both DVL3 (*P* = 0.036) and sFRP3 (*P* = 0.019) proteins. Significantly higher number of samples with moderate and strong staining had the signal located both in the cytoplasm and nucleus.

**Figure 2:**
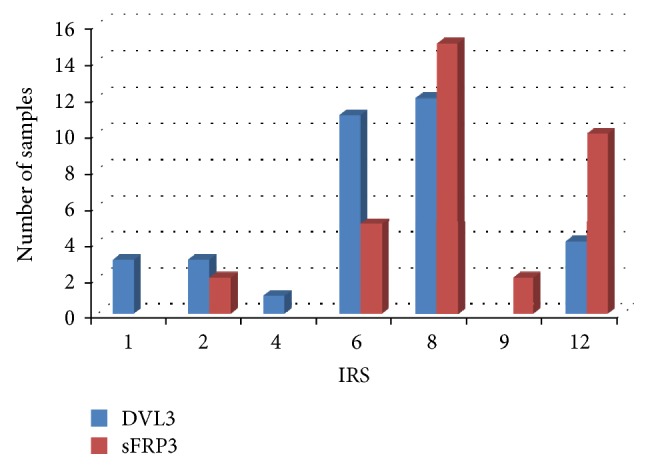
Graphs demonstrating IRS of DVL3 and sFRP3 protein.

The next step was to quantify the expression levels by using ImageJ (National Institute of Health, USA) program and introducing immunoreactivity score (IRS), a factor that best correlates with computational photoanalysis. IRS was calculated by multiplying the percentage of cells with a positive signal in the sample (PP score) with staining intensity (SI score). PP score was determined as follows: <1–20% positive cells = score 1; 20–50% = score 2; 50–85% = score 3; and >85% = score 4. SI score was assessed in three categories mirroring staining intensities: no staining or weak = score 1, moderate staining = score 2, and strong staining = score 3. IRS classification is based on the combination of information of PP and SI, and their product quantifies the observed amount of coloration. The IRS score in our study ranged from 1 to 12 (Figure 2).

Compatibility in the number of counted cells in the category of moderate signal strengths suggested potential correlation between the moderate expression levels of the two proteins. In order to test whether a correlation existed, the IRSes of DVL3 and sFRP3 were analyzed with Pearson's correlation and Student's *t*-test. Pearson's coefficient gave values of *r* = 0.3111 and *t* = 0.191 which corresponded to *P* value 0.40 > *P* > 0.25. These results suggest that there was no statistically significant correlation between DVL3 and sFRP3 expressions in glioblastoma.

**Figure 3:**
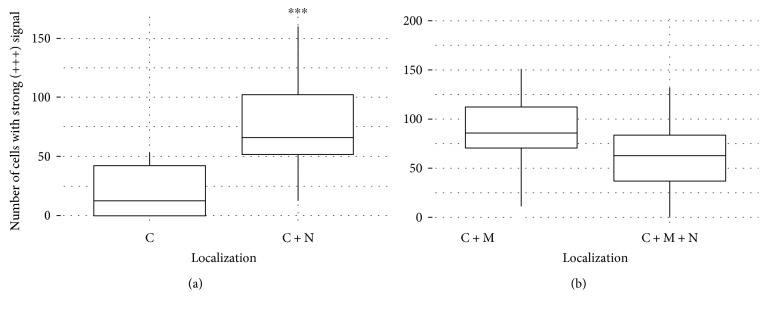
(a) Diagram of distribution of cells with strong DVL3 signal regarding localization (C and C+N). The graphs showing Wilcox rank of *W* = 241 and *P* value *P* = 6.33 × 10^−5^. ∗∗∗ denotes statistical significance. (b) Diagram of distribution of cells with strong sFRP3 signal regarding localization (C+M and C+M+N). The graphs showing Wilcox rank of *W* = 144 and *P* value *P* = 0.1903.

Furthermore, we investigated the difference in expression levels and the distribution of the signal regarding subcellular localization of the investigated proteins. When we investigate IRS of DVL3 and localizations on our total sample, the differences in the distributions were significant (*P* = 0.01). High IRS values were more frequent when the signal was localized in both cytoplasm and nucleus. Therefore, we analyzed the distribution of the signal with the nonparametric Mann–Whitney *U* test (Wilcox rank-sum test). We wanted to test the hypothesis that patients with the largest proportion of cells with strong expression have DVL3 protein localized in cytoplasm and nucleus and vice versa that patients with the largest proportion of cells with weak and moderate expression have DVL3 exclusively in the cytoplasm. The results showed Wilcox rank of *W* = 241 and *P* value of *P* = 6.33 × 10^−5^ (Figure 3(a)). These results indicate that patients with the largest proportion of cells with strong expression will almost certainly have signal localized both in the nucleus and cytoplasm, while the patients with the largest proportion of cells showing weak and moderate expression will have the signal limited to the cytoplasm. Glioblastomas with strong expression of DVL3 will significantly more often have the protein in the nucleus.

To analyze the differences in quantity distribution of sFRP3 to localization, we compared IRS of sFRP3 to different localizations. The results showed that the differences were marginally significant (*P* = 0.053). Exclusive cytoplasmic localization of sFRP3 showed higher IRS values. We also employed the nonparametric Mann–Whitney *U* test to sFRP3 values. We wanted to test if patients with the largest proportion of cells showing strong expression have sFRP3 localized at the membrane and in the cytoplasm and also if patients with the largest proportion of cells with weak and moderate expression have simultaneous localization of sFRP3 in all three cellular compartments, the membrane, cytoplasm, and nucleus. The results showed Wilcox rank of *W* = 144 and *P* value *P* = 0.1903 (Figure 3(b)). These numbers did not establish significant difference in the distribution of the expression strength of sFRP3 on the localization.

Finally, we investigated the association of epidemiological characteristics and anatomical site of the glioblastoma patients to DVL3 and sFRP3 expression levels and localizations. The relationship between IRS and sex was analyzed, but no significant difference was observed with respect to the sex of the patients. Both proteins demonstrate uniform expressions and localizations in glioblastoma cells in both sexes. To investigate the differences between the patients' age and expression and localizations of the proteins, we divided our sample into two age groups, younger than 56 and older than 56. Differences between age groups and levels or localizations of DVL3 and sFRP3 in glioblastoma were also not significant. Only one patient was aged below 21. We also looked into location of tumor, in respect to hemispheres, but the tumors we investigated were adult hemispheral GBM's and not located in the midline structures. Available glioblastoma samples were also tested for the presence of IDH1 R132H and ATRX mutations. Only one sample showed positive staining for IDH1 mutation. Five out of 10 samples (50%) available for the analysis showed ATRX protein loss. We also tested tumors diagnosed as recurrent to the expression levels of both proteins and found that all recurrent cases showed moderate and strong sFRP3 expression, while DVL3 was moderately expressed in 78% of recurrent glioblastoma. Remaining 22% showed lack of DVL3 expression. We also noticed that IRS score for sFRP3 was marginally associated to the recurrent cases (*P* = 0.087).

## 4. Discussion

During the past few years, a significant progress has been made in understanding the biology of glioblastoma formation [5, 38]; nevertheless, molecular events relevant for the development and progression of this tumor are still not fully understood [5, 6]. The cells of origin of gliomas, whether potentially astrocytes, glial precursors, or stem cells, are the subject of intense investigation as well. In a manner consistent with the cancer stem cell hypothesis, there is considerable evidence that only minor populations of cells in primary gliomas are capable of forming a tumor. Based on their biological characteristics, glioblastomas are in their cell composition, as well as genetically and pathologically, a very heterogeneous group of tumors. Molecular heterogeneity particularly lies in expression patterns of transcriptional regulators, tumor-suppressor proteins, and kinase mutations [39–41].

Aberrant Wnt signaling is responsible for the formation of a number of tumors in humans including medulloblastoma and glioblastoma [42–47]. The present study investigated the involvement of two Wnt signaling pathway proteins, DVL3 and sFRP3, in glioblastoma and demonstrated for the first time their expression levels and relationships. Quantitative evaluation of immunohistochemical analysis results can be difficult and needs to be objective. In order to quantify the expression levels, we evaluated our results by introducing immunoreactivity score (IRS). This score is improving the quantification of immunohistochemical staining [48, 49] making it more objective.

The results of this study showed that the majority of investigated samples had moderate levels of expression for both DVL3 (52.4%) and sFRP3 (52.3%). This compatibility in the expression levels suggested potential correlation between the two proteins. It has been indicated that sFRPs and DVLs can interact (STRING—known and predicted protein-protein interactions; http://string-db.org/). However, quantitative evaluation using IRS showed that there was no statistically significant correlation (*r* = 0.3111; *t* = 0.191; 0.40 > *P* > 0.25) between DVL3 and sFRP3 expressions in glioblastoma. It seems that the proteins are destined for different functional regulations.

In our study, we demonstrated that glioblastomas had high content of moderate (52.4%) and strong (23.1%) expression levels of DVL3 protein. Subcellular localization of the protein revealed that DVL3 was localized in cytoplasm in 53% of glioblastoma, while 44% of our total sample coexpressed the protein in the cytoplasm and nucleus. The differences in the IRS values and subcellular distributions were significant (*P* = 0.01). Moreover, the nonparametric Mann–Whitney *U* test (Wilcox rank-sum test) showed that patients with the largest proportion of cells with strong expression will almost certainly have signal localized both in the nucleus and cytoplasm, while the patients with the largest proportion of cells showing weak and moderate expression will have the signal limited to the cytoplasm. Glioblastomas with strong expression of DVL3 will significantly more often have the protein in the nucleus (*P* = 6.33 × 10^−5^) [49]. Li et al. [50] investigated the expression of Dishevelled in glioma and found that protein levels increased with the pathologic grade of glioma, so glioblastomas showed the highest levels in their study. These findings are compatible to ours. It is not unusual to find the DVL3 expressed in the nucleus, since it has been shown that it can play different cellular roles. The dynamics of DVL localization is regulated by its two sequences: nuclear localization signal (NLS) and nuclear export signal (NES) which are responsible for protein shuttling into and out of the nucleus [29–31]. This signal distribution corresponds to the different roles that DVL3 plays in cytoplasm and in the nucleus. In the cytoplasm, it binds to AXIN and disables beta-catenin's degradation, while in the nucleus it interacts with *β*-catenin and acts as a transcription factor [51]. Dishevelleds are considered to be key regulators that rescue cytoplasmic beta-catenin from degradation. When AXIN is recruited to the plasma membrane by DVL, AXIN can no longer be a part of beta-catenin destruction complex, so the complex cannot be formed resulting in the cytoplasmic accumulation of beta-catenin and its consequent transfer to the nucleus.

It has been shown by several authors and in our own previous studies [21, 42, 52] that beta-catenin shows upregulation and nuclear expression in glial tumors.

Increased expression of DVL3 protein could result in strengthening its transcriptional activity and consequently stimulating activity of Wnt signaling. Localization of DVL3 protein in the nucleus could be an indicator of poor prognosis. Gan et al. [51] examined several colon cancer tissue sections and reported that DVL3 appears to be accumulated at high levels (36%) in the nuclei of the cancer cells.

In 56% of cases with cytoplasmic expression, the protein was located in close proximity to the cell membrane which can be explained with the known fact that DVL3 can be engaged in the membranous complex pulling out AXIN from the beta-catenin destruction complex, thus preventing beta-catenin's degradation and elevating its cytosolic levels. The diversity of the signal localization could certainly be attributed to the heterogeneity of malignant tissue, as well as the fact that both investigated proteins in the cell perform multiple functions and interact with dozens of proteins.

Changes in DVLs have been reported in various tumor types, including lung, prostate, breast, cervical squamous cell carcinoma, and gliomas [53–58]. The functional consequences of the DVL family protein expression in tumor formation are inadequately explained and the data reported are contradictory. The majority of reports [54, 57] indicate DVL overexpression and amplification, but there are also reports on gross deletions of DVL loci [59, 60]. The overexpression of DVL contributed to the invasion of glioma cells [52, 61]. In addition, a correlation between the expression of DVL and the quantity of nuclear beta-catenin has also been established [52, 61]. The accumulation of nuclear beta-catenin induces EMT by activating repressor of E-cadherin, Snail, and Slug [50].

Our expectations on sFRP3 downregulation in glioblastoma were not proved. Although at first sFRP3 has been assigned a role in the inhibition of the Wnt signaling [62], there are many consistent reports that it is upregulated and can activate the pathway in tumor progression and metastasis [25, 32, 63–66]. Our findings demonstrate that in the majority of glioblastomas, sFRP3 was moderately (52.3%) and highly (36.0%) expressed, the levels being higher than the positive controls that we employed. The analysis of sFRP3 protein's subcellular distribution showed that it was localized in the cytoplasm in 94%. Colocalization in the cytoplasm and nucleus was observed in 50% of samples with the moderate and strong expression levels. The signal was also detected along the cell membranes of each sample which is not surprising considering its role as antagonist of Wnt signaling cascade competing with other antagonists for binding sites on Wnt and Fz receptor.

The results of our analysis of the differences in quantity distribution of sFRP3 as denoted by IRS score to localization showed that the differences were marginally significant (*P* = 0.053). Exclusive cytoplasmic localization of sFRP3 showed higher IRS values. When we tested our results employing Mann–Whitney *U* test (Wilcox rank-sum test), no significant difference in the distribution of the expression strength of sFRP3 on the localization were established (*P* = 0.1903), indicating that the intensity of sFRP3 expression was not confined to any subcellular compartments in particular [49]. sFRP3 represents a modulator, crucial for controlling Wnt signaling with a wide diapason of biological functions [11, 34, 67].

Epigenetic silencing of sFRP3 has been described in medulloblastomas [67, 68] as well as in melanoma tumor and cell lines [69] and glioblastoma cell lines [17]. Furthermore, there are also studies reporting the loss of sFRP3 alleles. Deletion of sFRP3 on locus 2q31-33 is commonly found in breast cancer, colorectal cancer, neuroblastoma, and lung cancer [25]. On the other hand, there is a number of novel studies reporting that sFRPs can also activate Wnt signaling in specific circumstances [32, 67] and many tumors investigated overexpressed sFRP proteins [32, 33]. Xavier et al. [67] showed that sFRP1 has a complex role within the regulation of the Wnt signaling. It can act as an inhibitor or an enhancer, depending on the cellular context, the concentration, and Fz receptor expression patterns. Its activity can be described as biphasic [11, 70]. The abovementioned studies are a good illustration of the complexity of signaling and diversity of biological functions of sFRP family proteins. It seems that similar to developmental gradients [71], specific spatiotemporal dynamic expression of sFRP3 is playing a role in glioblastoma too.

Our study may contribute to better understanding of glioblastoma molecular profile. It opens doors for future mechanistic investigations on DVL3 and sFRP3 roles in glioblastoma.

## Figures and Tables

**Table 1:** Percent of counted cells showing different levels of DVL3 and sFRP3 expression.

Signal strength	0/+	++	+++
DVL3	24.5%	52.4%	23.1%
sFRP3	11.7%	52.3%	36.0%

**Table 2:** Demographic variables, expression levels, and localizations of DVL3 and sFRP3 proteins in glioblastoma samples. Protein intensities are represented as percent of stained cells.

Patient number	Expression of DVL3 [%]			Cellular localization	Expression of sFRP3 [%]			Cellular localization	IDH1	ATRX	Intracranial localization	Sex	Age
	0/+	++	+++		0/+	++	+++						
1	18.5	42.5	39.0	C, N	69.5	30.5	0	C, M, N	ND	ND	Parietal left	M	61
2	94.0	6.0	0	C	16.5	44.0	39.5	C, M, N	ND	ND	Parietal right	F	34
3 R	100	0	0	—	32.5	67.5	0	C, M	—	ND	Parietal right	M	68
4	19.5	50.0	30.5	C, N	2.5	63.0	34.5	C, M	—	Retained	Temporooccipital right	F	54
5	6.5	84.5	9.0	C	0	56.0	44.0	C, M	—	ND	Temporal left	M	77
6	9.5	65.0	25.5	C	7.5	55.0	37.5	C, M, N	ND	ND	Parietal right	F	62
7	22.5	50.5	27.0	C, N	11.0	52.5	36.5	C, M, N	ND	ND	Parietal left	F	37
8	16.5	70.5	13.0	C, N	0	34.0	66.0	C, M, N	—	Retained	Temporal left	M	60
9	2.5	71.0	26.5	C	0	24.5	75.5	C, M	ND	ND	Frontotemporal left	M	72
10 R	16.0	78.0	6.0	C, N	22.0	75.0	3.0	M	ND	ND	Parietal left	F	70
11	9.5	20.5	70.0	C, N	0	40.5	59.5	C, M	—	ND	Frontal left	M	71
12	29.5	44.5	26.0	C	8.0	73.0	19.0	C, M, N	ND	ND	Occipital right	M	70
13	11.0	49.0	40.0	C, N	0	43.0	57.0	C, M	—	Loss	Parietal right	M	55
14 R	0	80.0	20.0	C	17.0	29.5	53.5	C, M	+	ND	Temporal left	M	31
15	3.5	16.5	80.0	C, N	4.0	58.0	38.0	C, M	—	Retained	Parietal left	F	56
16	14.0	61.5	24.5	C	0	3.5	96.5	C, M	ND	ND	Frontal right	F	74
17	18.5	78.0	3.5	C	9.0	71.5	19.5	C, M	—	Retained	Temporal right	F	56
18	6.0	74.5	19.5	C	6.5	56.5	37.0	C, M, N	ND	ND	Temporal right	F	53
19	5.0	63.0	32.0	C, N	8.5	68.0	23.5	C, M, N	ND	ND	Occipital left	M	36
20 R	7.5	74.0	18.5	C	0	15.5	84.5	C, M, N	—	Loss	Temporooccipital right	M	38
21 R	19.0	57.0	24.0	C, N	4.5	58.0	37.5	C, M	ND	ND	Temporooccipital left	F	62
22	63.5	36.5	0	C	0	36.5	63.5	C, M, N	—	ND	Frontoparietal left	F	68
23	61.0	39.0	0	C	0	96.5	3.5	C, M, N	ND	ND	Temporal left	M	67
24 R	22.0	56.5	21.5	C	14.0	37.5	48.5	C, M, N	—	Loss	Temporal left	F	59
25	19.5	68.0	12.5	C, N	73.5	26.5	0	M	ND	ND	Temporooccipital right	F	20
26	85.5	14.5	0	C	0	78.0	22.0	C, M, N	ND	ND	Temporal right	M	72
27 R	77.0	23.0	0	C	18.5	39.5	42.0	C, M	—	Loss	Frontotemporal left	M	31
28	33.0	67.0	0	C	6.0	78.0	22.0	C, M, N	—	Retained	Frontal right	M	54
29	3.5	17.5	79.0	C, N	10.5	35.5	54.0	C, M	ND	ND	Parietal right	M	70
30 R	20.0	47.0	33.0	C, N	0	74.0	26.0	C, M, N	ND	ND	Temporoparietal left	M	31
31	4.0	96.0	0	C	19.5	73.5	7.0	C, M, N	—	Loss	Frontal right	M	54
32	1.5	36.5	62.0	C, N	ND	ND	ND	ND	—	ND	Temporal left	F	75
33 R	2.0	58.5	39.5	C, N	ND	ND	ND	ND	—	ND	Frontal left and right	F	60
34	10.5	86.0	3.5	C	15.5	79.0	5.5	C, M	ND	ND	Temporal right	F	59

C = cytoplasmic; N = nuclear; M = membraneous; ND = not determined; − = not present; + = present; R = recurrent.
